# Genome Sequences of Zika Virus Strains Recovered from Amniotic Fluid, Placenta, and Fetal Brain of a Microcephaly Patient in Thailand, 2017

**DOI:** 10.1128/MRA.01020-18

**Published:** 2018-09-20

**Authors:** Thidathip Wongsurawat, Piroon Jenjaroenpun, Niracha Athipanyasilp, Bualan Kaewnapan, Nattawat Leelahakorn, Nasikarn Angkasekwinai, Wannee Kantakamalakul, Ruengpung Sutthent, David W. Ussery, Navin Horthongkham, Intawat Nookaew

**Affiliations:** a Department of Biomedical Informatics, College of Medicine, University of Arkansas for Medical Sciences, Little Rock, Arkansas, USA; b Department of Microbiology, Faculty of Medicine, Siriraj Hospital, Mahidol University, Bangkoknoi, Bangkok, Thailand; c Department of Medicine, Faculty of Medicine, Siriraj Hospital, Mahidol University, Bangkoknoi, Bangkok, Thailand; d Department of Physiology and Biophysics, College of Medicine, University of Arkansas for Medical Sciences, Little Rock, Arkansas, USA; University of Arizona

## Abstract

We present here the complete genome sequences of Zika virus strains isolated from aborted fetal tissue (brain and placenta) and amniotic fluid of a microcephaly patient in Thailand in 2017. The virus genomes that were sequenced have an average length of 10,807 nucleotides.

## ANNOUNCEMENT

Zika virus (ZIKV) is a linear, single-strand, positive-sense RNA virus of the family Flaviviridae. Due to an unprecedented number of infection outbreaks, ZIKV has become a global concern. The virus commonly spreads through mosquito bites and causes relatively mild symptoms in adults, but it has been shown to cause microcephaly in infants born to infected mothers. ZIKV is now spreading rapidly in Latin America, and in Asia, Thailand and the Philippines have had a high incidence of infection (1). The WHO confirmed the first microcephaly cases in Thailand in 2016, but to date, no genome sequences of ZIKV strains from Thailand have been reported. The genome sequences of ZIKV strains isolated from microcephaly cases in Asia may clarify the possible mechanisms for the transmission and pathogenesis of ZIKV. We report here the genome sequences of ZIKV strains isolated from aborted fetal tissue (brain and placenta) and amniotic fluid samples from a microcephaly patient in Thailand.

The patient was suspected of having ZIKV infection at 9 weeks of gestation. At 16 weeks, amniotic fluid tested positive for ZIKV, and the pregnancy was terminated at 17 weeks. An autopsy of the fetus demonstrated a head circumference of 12.5 cm, which does not reach the third percentile for this gestational age. RNA of ZIKV was detected in the brain and placenta. Viral RNA was extracted using a NucliSens kit (bioMérieux, France), and cDNA was synthesized using PrimeScript (TaKaRa, Japan). The whole-genome sequences of the ZIKV isolates were amplified using PrimeStar GXL (TaKaRa, Japan).

Three sequencing platforms were used for the different ZIKV isolates. The Ion Torrent platform was used for the brain isolate, and the Oxford Nanopore Technologies (ONT), Ion Torrent, and Illumina platforms were used for the amniotic fluid and placenta isolates. The read data generated for the isolates are listed in Table 1.

**TABLE 1:** Read information generated from three sequencing technologies

Sequencing technology	Isolate source	Total no. of bases	No. of reads	Mean length (bp)
Ion Torrent	Brain	9,276,530	45,701	202.98
Illumina	Amniotic fluid	2,019,009,458	8,807,177	229.25
Ion Torrent	Amniotic fluid	7,531,684	38,694	194.65
Oxford Nanopore	Amniotic fluid	224,694,525	139,132	1,614.97
Illumina	Placenta	1,853,740,947	9,189,342	201.73
Ion Torrent	Placenta	5,639,797	20,933	269.42

The Ion Torrent library was constructed using an Ion Xpress Plus fragment library kit (Life Technologies, USA), following the manufacturer’s instructions. The libraries were amplified on the OneTouch version 2 platform (Life Technologies, USA) and sequenced on an Ion Torrent PGM sequencer using the 316 chip. The Ion Torrent sequencing generated 9.2 million reads with an average read length of 202 bp. Genome assembly was performed using CLC software version 9.0 with default parameters (Qiagen, Germany).

Library preparation for the ONT sequencing followed the one-dimensional genomic DNA sequencing protocol SQK-LSK108 (ONT, USA). Sequencing of the library was performed on a single R9.4/FLO-MIN106 flow cell on the MinION Mk1B system. Base calling was performed using Albacore software version 1.2.3. Last, Illumina sequences were used to obtain high-quality genome sequences.

Library preparation for Illumina sequencing was performed with a KAPA HyperPlus kit (Kapa Biosystems, USA) following the manufacturer’s protocol. The libraries were sequenced using the MiSeq platform (Illumina, USA).

Raw reads were mapped using Burrows-Wheeler alignment with the MEM algorithm (BWA-MEM) and default parameters (2). Duplicates were removed using Picard MarkDuplicates version 2.9.2 with default parameters (https://broadinstitute.github.io/picard), and reads were realigned and consensus called using GATK version 3.7 (RealignerTargetCreator, IndelRealigner, SelectVariants, CombineVariants, and FastaAlternateReferenceMaker) with default parameters, except for SelectVariants, which used a modified command (-select “ABHet < 0.4 && DP > 100”) (3).

The reads were assembled using the ZIKV genome sequence available in GenBank (accession number KY272987; 98% identity) as a reference. The total size of the assembly was 10,807 bp. Genome annotation was performed with ViPR workbench (www.viprbrc.org) (4). Using a whole-genome comparison method (5), we phylogenetically analyzed the three ZIKV genomes in our recent report (6). The tree showed that the three genomes closely resemble the local Asian strains.

### Data availability.

The complete ZIKV genome sequences reported here have been submitted to GenBank and are publicly available under the accession numbers MG548660 (amniotic fluid), MG548661 (placenta), and MF996804 (brain).
